# Perfect meta-absorber by using pod-like nanostructures with ultra-broadband, omnidirectional, and polarization-independent characteristics

**DOI:** 10.1038/s41598-018-25728-7

**Published:** 2018-05-08

**Authors:** Yu-Sheng Lin, Wenjun Chen

**Affiliations:** State Key Laboratory of Optoelectronic Materials and Technologies, School of Electronics and Information Technology, Sun Yat-Sen University, Guangzhou, 510275 China

## Abstract

The on-chip perfect meta-absorber (PMA) is an important optical and thermal energy component in photovoltaics, thermal emitters, and energy harvesting applications. However, most reported PMAs rely on the complicated lithography techniques, which imposed a serious cost barrier on the development of practical applications, especially in the visible to near-infrared (NIR) wavelength range and at very large scales. Importantly, it is hard to realize PMA in the UV wavelength range by using current lithography techniques. In this article, we develop an ultra-broadband PMA by using natural lithography (NL) technique. The morphology of proposed PMA is randomly distributed pod-like nanostructures composed of a nanocomposite (Au/SiO_2_) covered a gold layer. It can be formed easily on Si substrate to function as an ultra-broadband, omnidirectional, and polarization-independent PMA by controlling the conditions of sputtering deposition and thermal annealing treatment. We experimentally realized an on-chip ultra-broadband PMA with almost 100% absorption spanned from UV-visible to NIR wavelength ranges. This cost-effective and high-efficiency approach would release the manufacturing barrier for previously reported PMAs and therefore open an avenue to the development of effectively energy harvesting, energy recycling, and heat liberation applications.

## Introduction

Recently, researches in plasmonic fields are rapidly maturing. One emerging field of them is taking advantage of unique electromagnetic properties to achieve perfect meta-absorber (PMA) due to the huge interest in the development of materials for harvesting solar energy. PMA is a material that completely absorbs incident light spanned broad wavelengths. There is no light either transmitted or reflected and the material surface appears black color to human eyes^1^. This emerged with the recent development of PMA is capable of angle-insensitive and polarization-insensitive^2–4^, which are important optical and thermal energy components for practical applications. The absorbed energy of light from PMA is generally converted to heat and/or photocarriers making it desirable for a widespread applications such as solar energy harvesting^5^, thermal emitters^6–9^, photon detectors^10–13^, photovoltaic cells^14,15^, photocatalysis^16,17^, spectroscopy^18–20^, biosensors^21–23^ and so on.

To date, most of the applied methods, including metamaterial nanoantennas^3,4,19^, perforated metallic films^24–26^, structured nanogratings^27–29^, and tapered nanostructures with gradient refraction index^30–32^ are along with the current advancements in nanofabrication techniques, it has made the fabrication of PMA easier and controllable. Despite the efficient absorption of light, the fabrication of these nanostructures requires the fine control of geometrical dimensions at the nanoscale achieved by using electron beam lithography (EBL), focused ion beam (FIB), and nanointerference lithography (IL)^33–35^. These techniques hinder their upscaling and practical use. The major problem of EBL, FIB and IL techniques is low throughput, which is not suitable for large-area fabrication. The alternative methods for large-area nanofabrication are nanosphere lithography (NSL)^19,30^ and nanoimprinted lithography (NIL)^3,36^. However, it is a challenge for batch fabrication of nanostructures due to the hard control of complicated synthesis processes in NSL and imprinting conditions in NIL. These methods are costly and suffer from a lack of flexibility. Furthermore, their absorbance is limited to a narrow bandwidth of spectrum, which makes their application for the requirement of broadband spectral impossible. To overcome the above problems, natural lithography (NL) technique is a relatively simple and cost-effective method to make large-area nanostructures from a substrate with metallic nanoparticles (NPs) on it. Using NL technique, the metallic NPs could be easily controlled their dimensions, densities, and distributions by modifying the metal deposition and thermal annealing conditions. The merits of NL technique are not only the metallic NPs could be easily controlled but also the metallic NPs show high absorption due to its localized particle plasmon resonance, i.e. Mie resonance^37–39^. The resonance of these metallic NPs embedded in different matrices has been extensively studied in the last decade and it is well known that the resonant bandwidth depends on the size, shape, density, and distribution of the metallic NPs. Since the excitation of the localized plasmon resonance of the metallic NPs, the highly dense nanocomposite gives rise to a very broadband absorption in the visible wavelength range^39^.

Here, we demonstrate design, fabrication, and characterization of a PMA in a stack of metal and nanocomposite by using directly sputtering deposition and thermal annealing treatment. The experimental results indicate our proposed PMA with pod-like nanostructures is ultra-broadband, omnidirectional and polarization-independent. The design of PMA has almost 100% absorptance spanning an ultra-broadband wavelengths from UV-visible to the near-infrared (NIR) spectral ranges. The fabrication technique of our PMA is relatively simple, cost-effective, and compatible with current industrial methods for mass production, which makes our design an outstanding candidate for high efficiency absorber materials.

## Results

**Figure 1:**
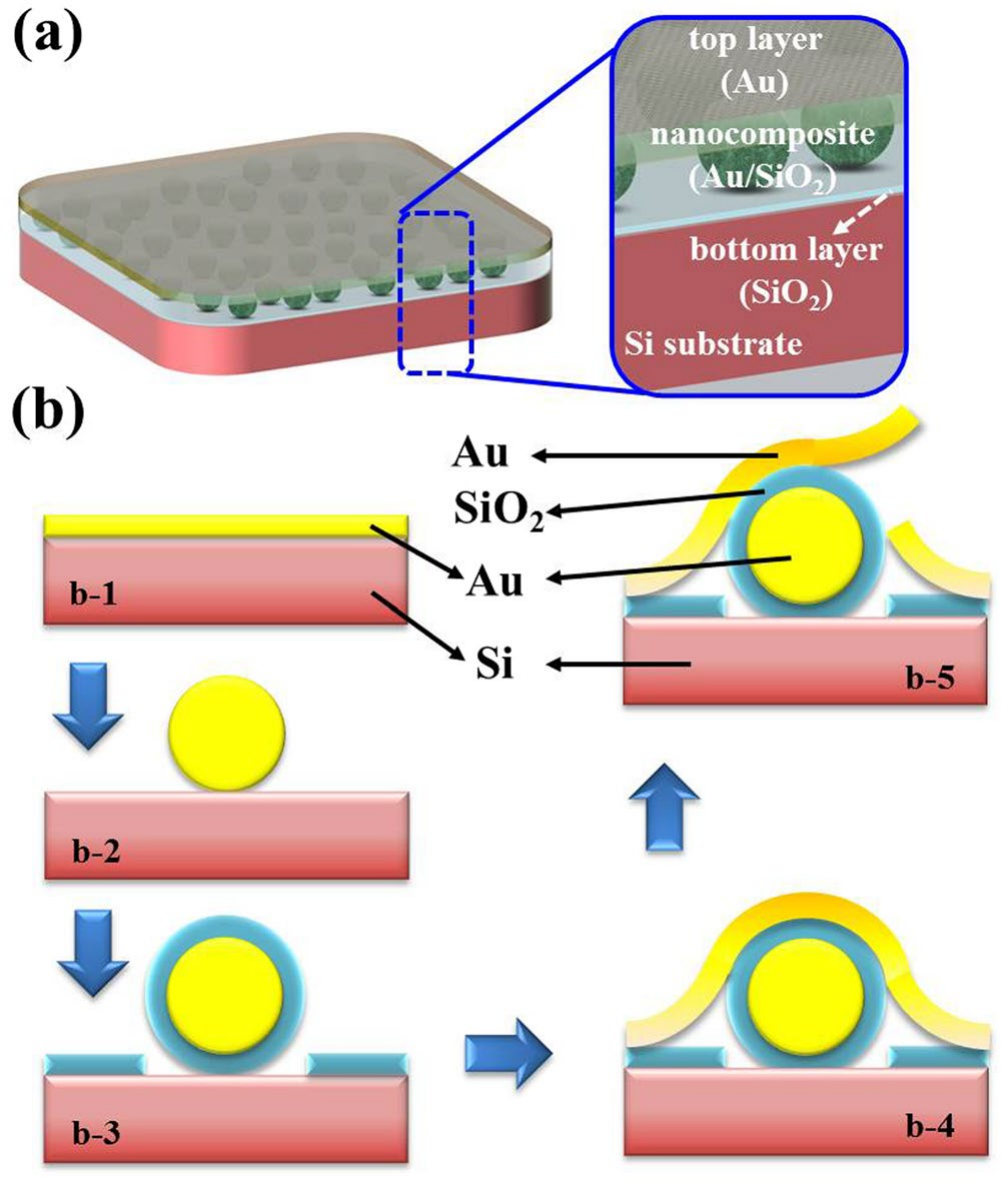
Schematic drawing of PMA and corresponding fabrication process. (**a**) The composition of PMA with pod-like nanostructures. The proposed pod-like nanostructures is composed of a SiO_2_ layer on Si substrate, an optical nanocomposite (Au/SiO_2_) layer and followed by a cover layer (Au) from bottom to the top. (**b**) Fabrication process of proposed PMA.

**Figure 2:**
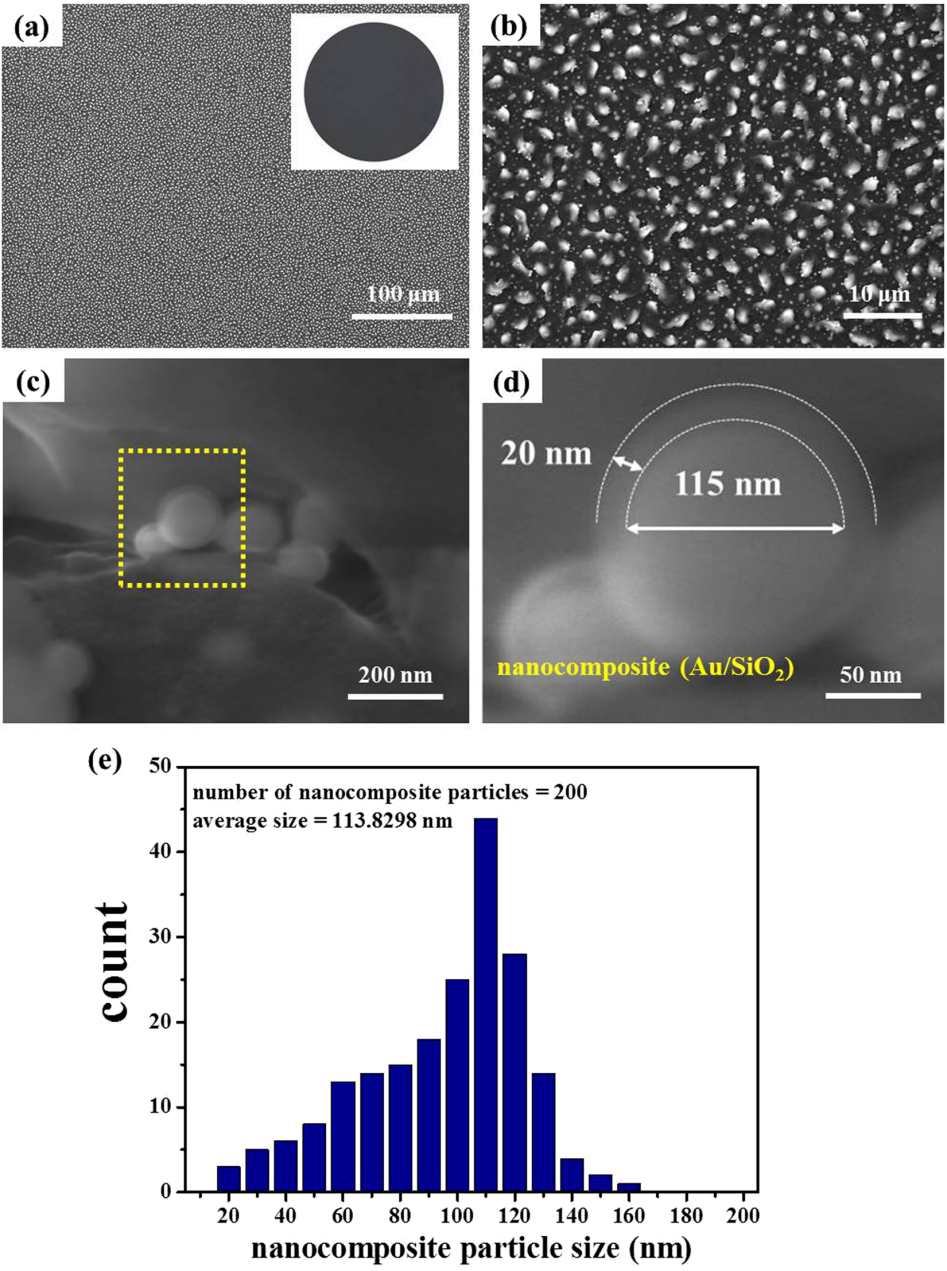
FESEM images of PMA. (**a**) FESEM image of pod-like nanostructures with an Au thin-film in 20 nm thickness at annealing temperature of 600 °C conditions, the insert image of (a) is the corresponding photography of full 4-inch PMA wafer. (**b**) FESEM zoom-in image of (a). (**c**) FESEM image of unit cell of pod-like nanostructures. (**d**) FESEM zoom-in image of yellow dash-square area in (c). The diameter of Au-NP is 115 nm and the thickness of SiO_2_ cladding layer is 20 nm. (**e**) The typical size distribution of nanocomposite particles embedded in the pod-like nanostructures. The average nanocomposite particle size is 114 nm.

**Figure 3:**
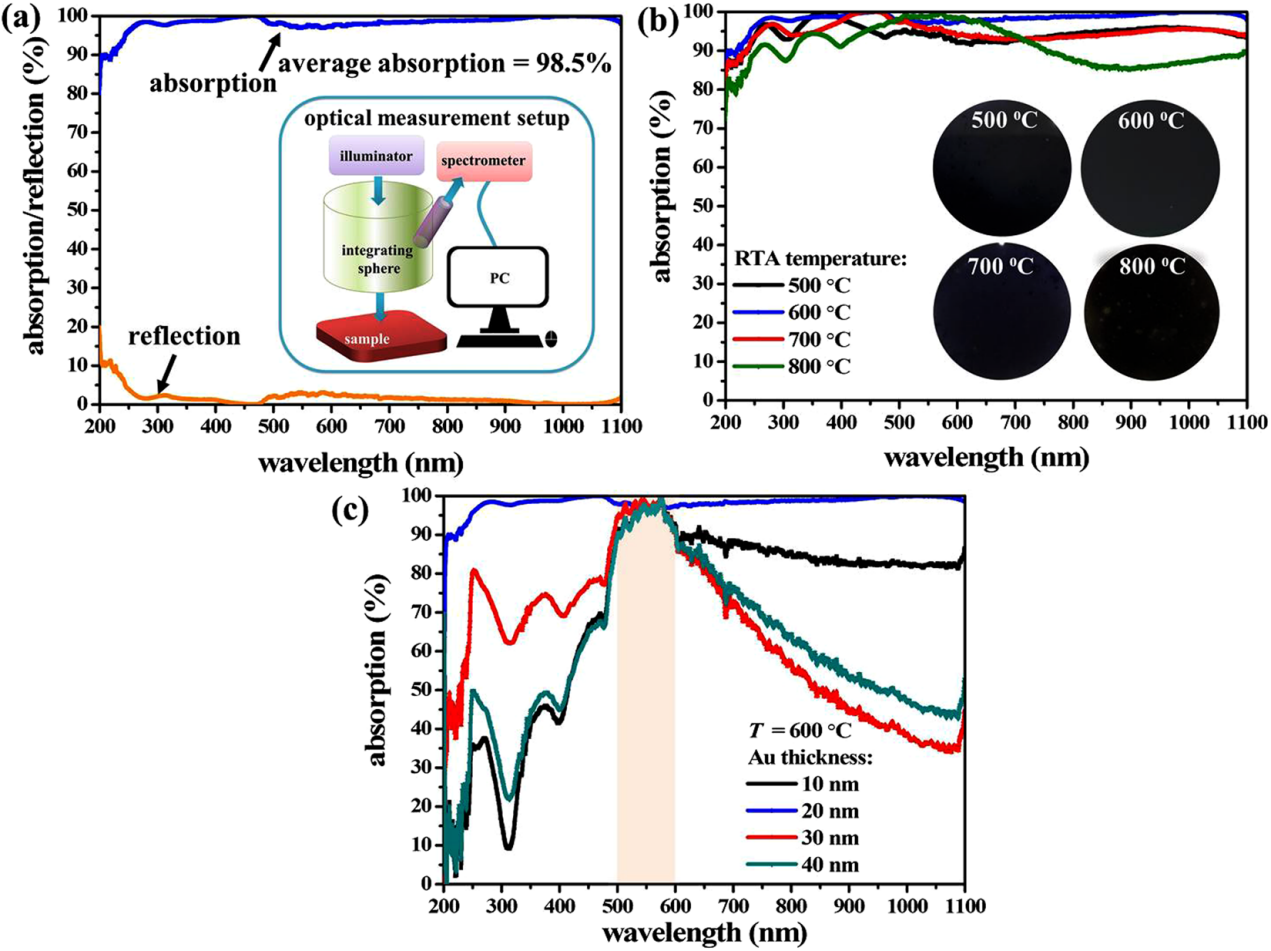
Reflection and absorption spectra of PMA. (**a**) Absorption spectrum of PMA with an Au layer of 20 nm thickness kept at 600 °C thermal annealing temperature. Insert image is the schematic drawing of optical measurement setup. (**b**) Absorption spectrum of PMAs with an Au layer in 20 nm thickness at different thermal annealing temperature. Insert images are the photographs for 500 °C, 600 °C, 700 °C, and 800 °C thermal annealing temperature, respectively. (**c**) Absorption spectra of PMAs with different Au thickness kept at 600 °C thermal annealing temperature. All optical measurements were measured at normal incidence.

**Figure 4:**
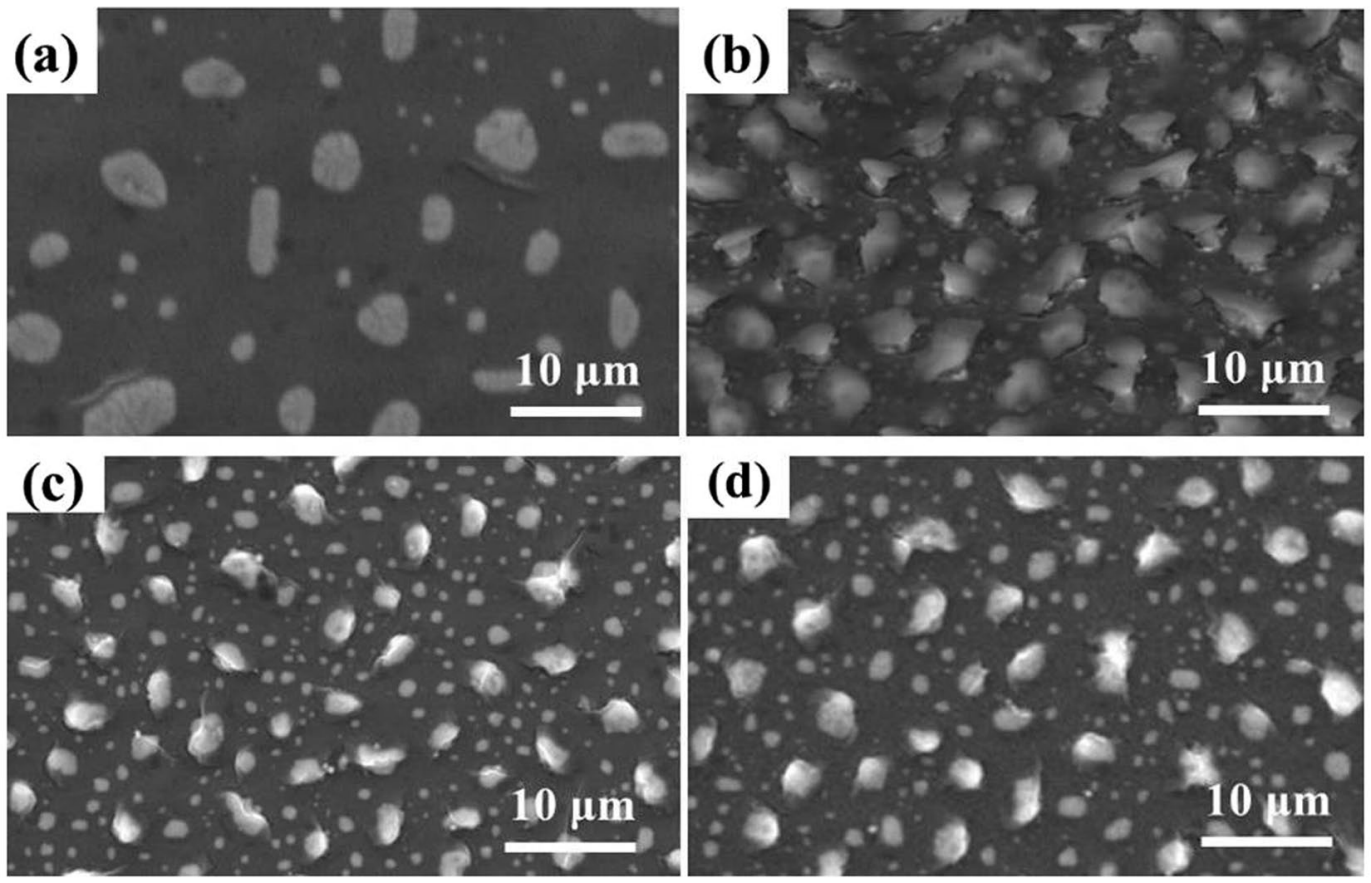
FESEM images of PMA with different thermal annealing temperature. Thermal annealing temperature is at (**a**) 500 °C, (**b**) 600 °C, (**c**) 700 °C, and (**d**) 800 °C, respectively (All Au layers are kept at 20 nm in thickness).

**Figure 5:**
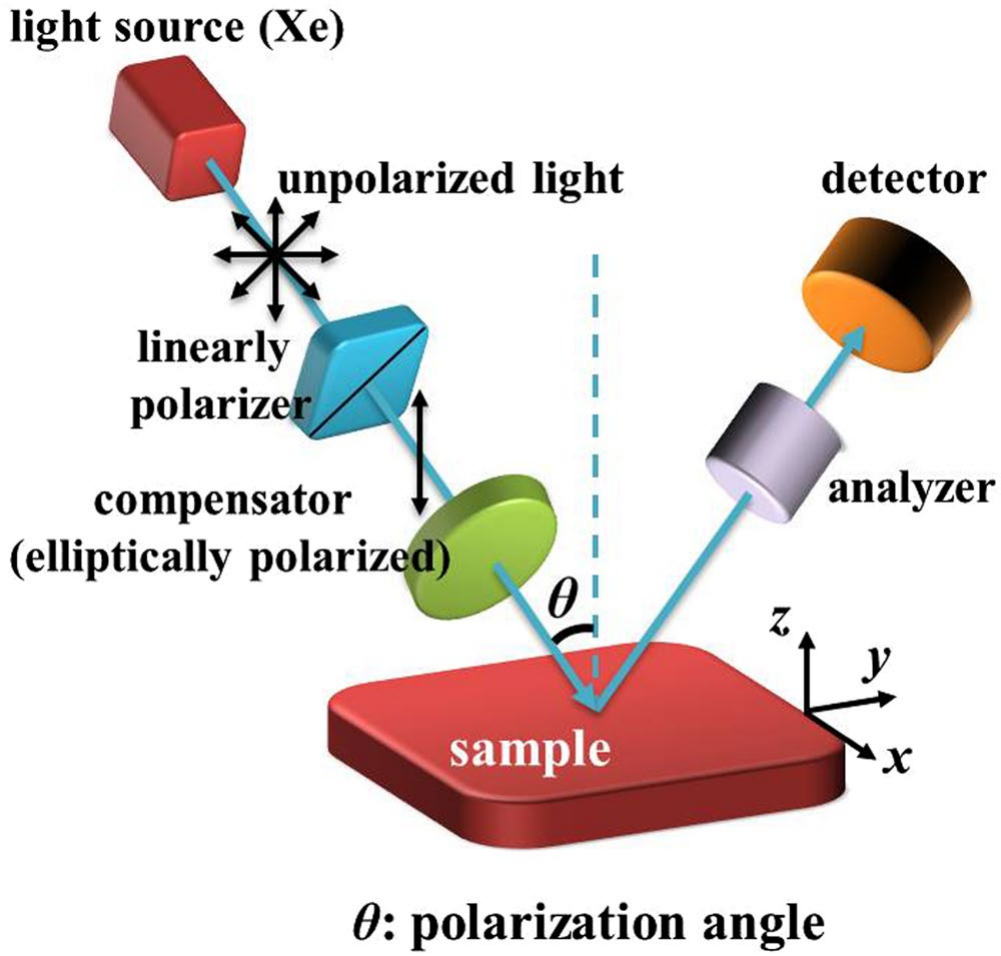
Schematic drawing of the reflected polarization measurement setup.

In this study, we combine directly sputtered Au layer and thermal annealing treatment processes to form the pod-like nanostructures on PMA surface for the development of a low cost and large-area manufacturing method. As illustrated in Fig. 1(a), the proposed pod-like nanostructures is composed of a SiO_2_ layer on Si substrate, an optical nanocomposite (Au/SiO_2_) layer and followed by a cover layer (Au) from bottom to the top. Among of these pod-like nanostructures, a SiO_2_ layer on Si substrate, a nanocomposite layer, and an Au layer are alike the bottom shell, beans, and top shell of the pod-like nanostructures, respectively. It is distinct from previously reported using three layered meta-absorber designs, i.e. a nanopatterned metasurface on a flat spacer and a metal ground plate layer^34–36^. Here, we adopted an inverted configuration by using sputtered Au layer and thermal annealing treatment processes to form the pod-like nanostructures on Si substrate directly. Figure 1(b) illustrates the fabrication process of the PMA by using NL technique to perform a large-scale fabrication. First, a 4-inch Si wafer with unintentional doping was used as the substrate. Afterwards, an Au layer in 20 nm thickness was sputtered on the cleaned Si substrate as shown in Fig. 1(b-1). Second, the sample was annealed at thermal temperature of 600 °C at 250 mTorr in Ar ambient to form Au nanoparticles (Au-NPs) on sample surface as shown in Fig. 1(b-2). Third, a SiO_2_ layer in 20 nm thickness was deposited on sample surface by using plasma enhanced chemical vapor deposition (PECVD) (Fig. 1(b-3)). Fourth, a top Au layer in 20 nm thickness was sputtered for the second time (Fig. 1(b-4)). This sputtering deposition of Au layer is the same conditions of Fig. 1(b-1). Finally, the sample was annealed at thermal temperature of 600 °C with the same process conditions of Fig. 1(b-2). Therefore, the second sputtered Au layer would be peeled off owing to the residual stress released after the sample exposed to ambient at room-temperature as shown in Fig. 1(b-5)^40,41^.

The plan-view field-emission scanning electron microscopy (FESEM) image reveals the morphology of the PMA with randomly sized pod-like nanostructures formed on the sample surface as shown in Fig. 2(a). The insert image of Fig. 2(a) is the corresponding photography of full 4-inch PMA wafer. The wafer surface is perfect black color. To magnify the FESEM image of Fig. 2(a), it is clear to be observed the pod-like nanostructures randomly distributed on PMA surface. Pod-like nanostructures are composed of nanocomposites covered by a continuous Au thin-film with crack patterns, while the nanocomposites are formed by Au-NPs covered with a SiO_2_ cladding layer on each Au-NP surface as shown in Fig. 2(c). In Fig. 2(d), it is a single nanocomposite particle (Au/SiO_2_). The diameter of Au-NP is 115 nm and the thickness of SiO_2_ cladding layer is 20 nm. The nanocomposite particle sizes on PMA surface are distributed from 20 nm to 160 nm and the average nanocomposite particle size is 114 nm as shown in Fig. 2(e).

The measured absorption and reflection spectra of our proposed PMA are illustrated in Fig. 3(a). The insert drawing of Fig. 3(a) is the schematic drawing of optical measurement setup. As shown in Fig. 3(a), the average absorption is 98.5% (almost 100%) measured at normal incidence from 200 nm to 1100 nm wavelength range. This strong absorption is due to the nanocomposites embedded in pod-like nanostructures. In the wavelength range from 200 nm to 1100 nm, the PMA exhibits a high absorbance of almost 100%. The absorption data (*A*) was obtained by measuring the specular reflectance (*R*) and using the principle of conservation of energy, which is expressed by *A* = 100%–*R*–*T*. Since the transmittance is completely canceled by the opaque substrate, the absorption was determined directly to be *A* = 100%–*R*. It means the high absorption obtained by the low reflection. In this study, the reason of super high absorption of PMA is attributed to the impedance matching of the sample to the vacuum, i.e. the unit cell of pod-like nanostructures can achieve zero reflection owing to that can have an impedance equal to the free space value which is indicated as $Z=\sqrt{\mu /\varepsilon }=1$^27,39,42^. The impedance matching of PMA with pod-like nanostructures results from electromagnetic optical resonance, which is induced by dipole/image interaction and causes an electromagnetic confinement between the pod-like nanostructures and thereby eliminates the reflection. This is the electromagnetic resonance since the circulating anti-parallel currents excited in the nanocomposites embedded in the pod-like nanostructures^27,43^. Afterwards, a strong enhancement of the localized electromagnetic field is established in the pod-like nanostructures and consequently no light is reflected back. Using this design of pod-like nanostructures with nanocomposites inside, it can sufficiently block the light from passing through along with the trapping of the light and suppressing the reflection. Eventually, the incident light will be obstructed completely and then realized the sample to have perfect absorption.

To demonstrate the influence of thermal annealing temperature to the absorption of PMA, the thermal annealing temperature of PMA changed while keeping the other parameter constant. We prepared four PMA samples kept the Au layer at 20 nm thickness and annealed at different thermal temperature of 500 °C, 600 °C, 700 °C, and 800 °C, respectively. The morphologies of these samples were characterized by FESEM as shown in Fig. 4, indicating that randomly distributed Au-NPs embedded in pod-like nanostructures. The optical absorption spectra of PMA samples are indicated in Fig. 3(b). The average absorption spectra are 92.6%, 94.8%, 98.5%, and 90.3% for thermal annealing temperature of 500 °C, 600 °C, 700 °C, and 800 °C, respectively, from the UV-visible to NIR wavelength ranges. The insert images of Fig. 3(b) are the photographs for PMA samples at different thermal annealing temperature. It is clear to be observed the surface colors of samples are black. Meanwhile, we also prepared another four PMA samples to investigate the influence of sputtering conditions to the absorption of PMA. These four samples were with nominal mass thicknesses of 10 nm, 20 nm, 30 nm, and 40 nm (estimated from the displayed deposition rate of the sputtering system) on unintentional doping and pre-cleaned Si substrates. The thermal annealing conditions were identical kept at 600 °C to expect the influence of sputtering conditions to the super absorption characterizations. The absorption spectra of these samples were measured as shown in Fig. 3(c), indicating the average absorption spectra of PMA samples are 95.4%, 95.5%, 97.6%, and 98.3% at the 500 nm to 600 nm wavelength range for PMA samples with different Au sputtered thickness. The average absorption is almost 100% for the condition of Au in 20 nm thickness from UV-visible to NIR wavelength range, i.e. λ = 200–1100 nm.

The role of electromagnetic resonance in the ultra-high absorption of PMA with a sputtered Au layer in 20 nm thickness and thermal annealing at 600 °C conditions is further confirmed by measuring the optical spectra with polarized light. The polarized reflection spectra of PMA were conducted by using spectroscopic ellipsometer (SE). SE technology plays a key role in the determination of optical parameters and film thickness by virtue of its nondestructive and accurate measurement. In order to measure the polarized reflectance for the wavelengths between UV-visible to NIR spectral ranges, we used a Xenon-lamp illuminator. The measurement setup of polarized reflection spectra of PMA is shown in Fig. 5.

**Figure 6:**
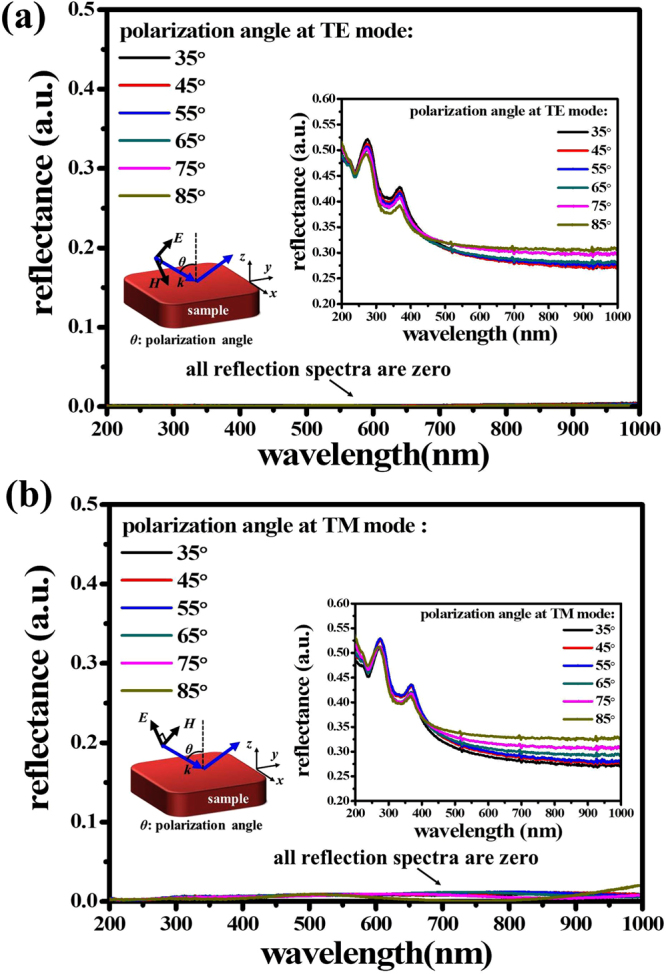
Polarized reflectance of PMA at different polarization angle. The reflection spectra were measured at (**a**) TE and (**b**) TM modes, respectively. The insert schematic drawings and reflection spectra of (**a**) and (**b**) are the polarization states of incident light and the bare Si substrate as a reference measured at TE and TM modes, respectively.

The experimental results of PMA measured at TE and TM modes in the angles ranging from 35° to 85° are shown in Fig. 6. The polarization state of incident light for both modes are also indicated in the insert schematic drawings of Fig. 6(a and b), respectively. For comparison, we also prepared a bare Si substrate as a reference to measure its reflectance at TE and TM modes shown in the insert figures of Fig. 6(a and b), respectively. In Fig. 6(a), the measured reflection spectra of PMA at TE mode are almost zero from the incident angle of 35° to 85°. It is the same optical responses for the PMA measured at TM mode as shown in Fig. 6(b), which reflection intensities of spectra are lower than 0.01. It is very close to the ideal perfect absorber. All reflection intensities of spectra of bare Si substrate and our proposed sample are a little variation for different polarization angle. The reason of these variations at TE and TM modes is the ambient effect. All reflection spectra were measured in an exposure area with surrounding air not in vacuum environment. The incident polarization light is easily affected by surrounding air. Even then, the reflection intensities of spectra of PMA are quite lower and exhibits omnidirectional and polarization-independent characteristics.

**Figure 7:**
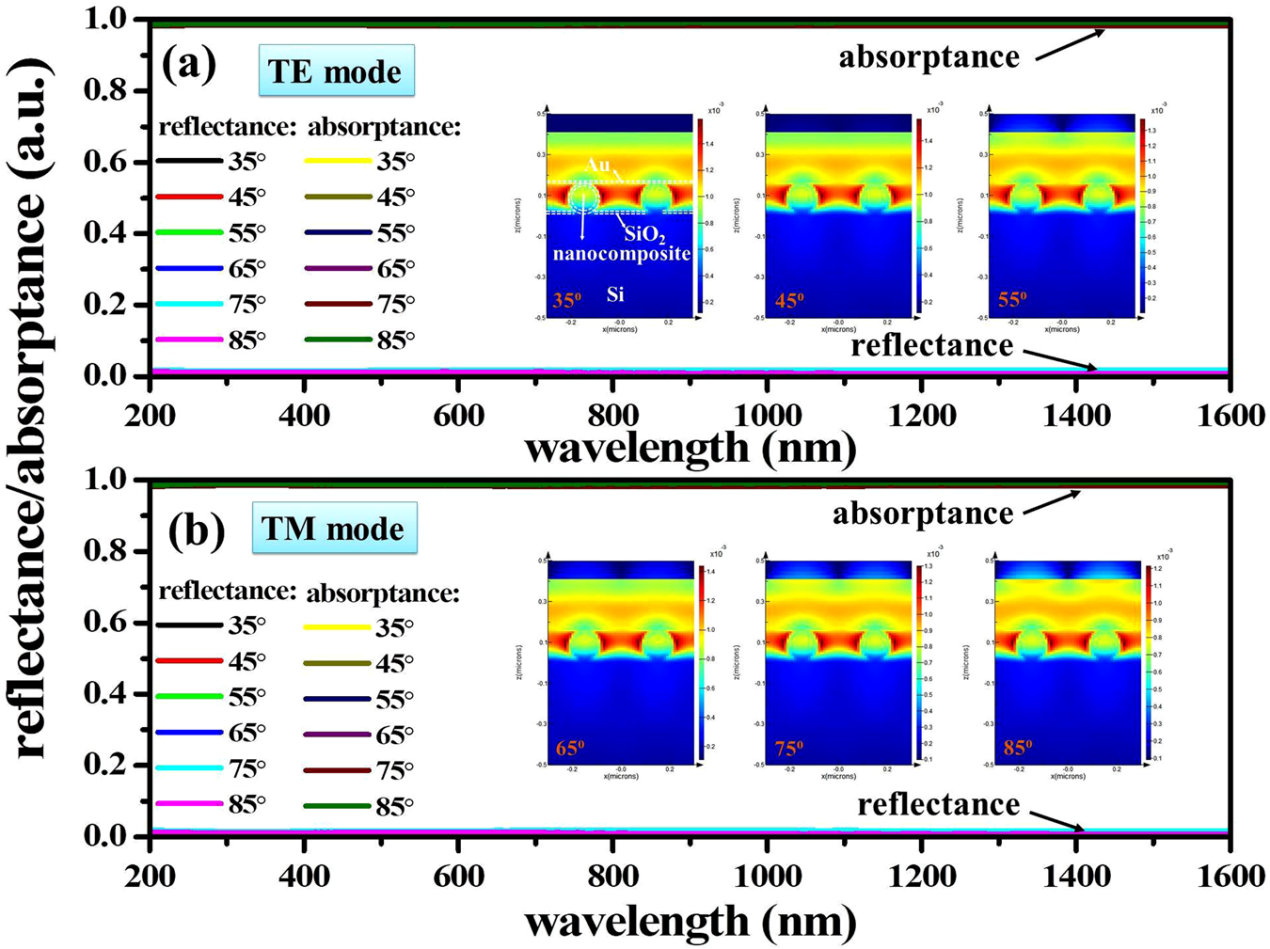
Simulated reflection and absorption spectra of proposed PMA at different polarization angle. The reflection and absorption spectra of PMA with pod-like nanostructures were simulated at (**a**) TE and (**b**) TM modes, respectively. The insert images of (**a**) and (**b**) are the electric field distributions from incident polarization angle from 35° to 85°, respectively.

To clarify the physical mechanism of the perfect absorption from pod-like nanostructures, the numerical simulations using the finite-different time-domain (FDTD) method were performed to calculate the reflection and absorption spectra and the electromagnetic field distributions with different incident angle of either TE or TM polarization as shown in Fig. 7. In the simulation, PMA with pod-like nanostructures were simulated by employing aperiodic nanocomposites covered a 20-nm-thickness Au thin-film on Si substrate with a 20-nm-thickness SiO_2_ thin-film atop to form the pod-like nanostructures. These nanostructures were excited by using a plane wave incident from the top side of sample. The mesh size is 2 nm in z-direction (perpendicular to the sample surface) and 5 nm in x and y directions and the simulation area is 600 nm × 600 nm. The reflection spectra were calculated computationally agrees well with experimental results. The observed ultra-broadband absorption is innate properties of random nanocomposites of pod-like nanostructures to facilitate the omnidirectional, and polarization-independent characteristics. The absorption of PMA with pod-like nanostructures is highly broadband and extends further into NIR spectrum. Investigating the field profile can be insightful in understanding field penetration as shown in the insert images of Fig. 7(a and b) represented the field profiles in the electric field distributions at different polarization angle from 35° to 85°, respectively. Therefore, the simulation results of PMA with pod-like nanostructures are deduced that field penetration from nanocomposites to bottom layers is negligible. We attribute the ultra-broadband absorption of proposed PMA to two effects. The first is the hybrid plasmonic coupling between the broad Mie resonance of Au-NPs originated from the randomly size distribution as shown in Fig. 2(e). The second is the plasmon polariton of the top metal film (Au) induced plasmonic resonance in the pod-like nanostructures since the interaction within Au-NPs plasmon resonances in the nanocomposites. It gives rise to an ultra-broadband absorption of PMA. The optical responses of PMA are clearly identical for both TE and TM modes due to the electromagnetic field in the pod-like nanostructures are isotropic for a wide range of incident angle. The overall absorption is about 100% in a broad range of the spectrum, i.e. from UV-visible to NIR wavelength range, which shows the potential of using this proposed PMA as a solar absorber. Furthermore, the angular independence of PMA for both polarizations indicates that our PMAs are effective solar absorbers with an ultra-broadband, omnidirectional, and polarization-independence, in contrast to known most of plasmonics exhibit angle-sensitive or polarization-sensitive behavior caused from the symmetric structure or asymmetric structure with periodic configurations, respectively^2–4^. According to the above mentioned, PMA with the pod-like nanostructures indeed exhibits the omnidirectional and polarization-independent characteristics. Such stable optical absorption performance with wide incident angle is expected to create new regimes of optical/thermal physics for impacting a broad range of energy technologies ranging from photovoltaics, to thin-film thermal absorbers/emitters, to optical-chemical energy harvesting.

## Discussion

The proposed PMA is designed to absorb most of light in the pod-like nanostructures. This development is a simple, low-cost and large-area method to fabricate a PMA with an ultra-broadband, omnidirectional, and polarization-independent super absorption. The optical absorption is almost 100% from UV-visible to NIR wavelength ranges. The electromagnetic resonance was supported in the optical patch antenna constructed by randomly distributed pod-like nanostructures on PMA surface. Importantly, the proposed fabrication technique based on the direct sputter deposition and post thermal annealing treatment is simple, cost-effective, and potentially scalable for mass production. Therefore, it will open an avenue to widespread applications, such as solar energy harvesting, thermal emitters, photodetectors, light emitting panels, biosensors and so on.

## Methods

### Sample fabrication

The proposed structurally PMA with pod-like nanostructures were fabricated by using NL technique. The 4-inch Si wafer with unintentional doping was sequentially sonicated in acetone, isopropyl alcohol (IPA), and deionized (DI) water for 10 min. Au layers with different thickness were directly deposited on cleaned Si substrates using a Quprum Q150T ES sputtering system. The deposition conditions were direct-current Ar plasma at 250 mTorr, gas purity of 99.995%, and discharge current of 50 mA. The sputtering time was controlled to obtain the target thickness. A PECVD SiO_2_ layer of 20 nm thickness was deposited on the sample surface by using OXFORD Instruments PlasmaPro 800plus System. The top Au covered layer of PMA was prepared by the same sputtering system and repeated the deposition process. The morphologies and particle sizes of the produced structures were measured by FESEM (Zeiss SUPRA 60).

### Characterization

A tungsten halogen lamp with a gold reflector providing a wavelength range of 200–1100 nm and a peak power of 150 W was used as a light source. The optical properties of the PMAs were characterized by using a UV-NIR spectrometer (Maya2000 Pro., Ocean Optics Ltd. Co.) connected to a microscope together with an integration sphere under a spectral range of 200–1100 nm. All measurements were directed perpendicular to sample and the reflected light from the microscope fed to a UV-NIR spectrometer and the data was collected by interfacing the spectrometer with PC. The measured reflections were referenced to an Al mirror to determine the absolute reflectivity. The polarized reflection spectra of PMA were conducted by using HORIBA UVISEL2 spectroscopic ellipsometer (SE). SE is a nondestructive, contactless and very reliable technique extensively used to investigate the optical properties of natural and artificial materials including metallic NPs. It enables us to measure the polarized reflectance at TE and TM modes also which further helps in understanding separately the behavior of various dipolar resonances in metallic NPs. The incidence angle can be varied from 35° to 85° and available wavelength range for measurement is 200 nm to 1100 nm.
